# The AGT M235T variant and renin–angiotensin–aldosterone system parameters in a predominantly hypertensive elderly black South African cohort: a hypothesis-generating descriptive case series

**DOI:** 10.1007/s11033-026-12994-2

**Published:** 2026-10-23

**Authors:** Christa Johanna Grobler, Wilna Oldewage-Theron, Joseph Musonda Chalwe

**Affiliations:** 1 Department of Health Sciences, Vaal University of Technology, Vanderbijlpark, 1900 South Africa; 2 Faculty of Science, Nelson Mandela University, Gqeberha, South Africa; 3 ARC/DALRRD/UFS Co-Chair: Impact Assessment of Climate-Smart Interventions, Department of Sustainable Food Systems and Development, University of the Free State, Bloemfontein, South Africa; 4 Nuclear Medicine Research Infrastructure, Capital Park, Pretoria, South Africa; 5 Department of Nuclear Medicine, Faculty of Health Sciences, University of Pretoria, Pretoria, South Africa

**Keywords:** M235T, Renin–Angiotensin–Aldosterone system, Black, Elderly

## Abstract

**Background:**

Hypertension (HTN) is a key cardiovascular risk factor, with its pathogenesis remaining incompletely understood. Blood pressure (BP) regulation involves the Renin–Angiotensin–Aldosterone System (RAAS), including the Angiotensin-converting enzyme (ACE), Angiotensin I (Ang I) and Angiotensin II (Ang II). The rs699 variant (M235T) may influence BP via the RAAS.

**Aim:**

This study aimed to evaluate the relationships between the rs699 genotypes, RAAS parameters, BP, Body mass index (BMI) and gender in a black elderly population.

**Methods:**

Data were collected from 48 participants aged ≥60 years, using World Health Organization (WHO) obesity guidelines and South African Hypertension Society (SAHS) 2019 standards for HTN categories. Statistical analysis was performed using IBM SPSS version 27 and Python 3.14.

**Results:**

Most participants possessed the rs699 variant (GG: 93.8%, AG: 4.2%). ACE levels were low in 64.6%, while Ang II was elevated in the same percentage. The majority were classified as hypertensive and obese. No significant associations were found between the rs699 variant and ACE, Ang I, Ang II, HTN, BMI or gender, though BMI showed sex-related differences in females.

**Conclusion:**

Our findings suggest no significant association of the rs699 variant on RAAS or cardiometabolic parameters within the study population, warranting cautious interpretation. These findings require validation in larger and more diverse populations, as meaningful statistical inferences cannot be drawn from the current data.

**Supplementary Information:**

The online version contains supplementary material available at https://doi.org/10.1007/s11033-026-12994-2.

## Introduction

HTN is a leading global risk factor for morbidity and mortality, affecting more than one billion adults worldwide, yet its underlying mechanisms remain incompletely understood [1, 2]. Among the key pathways involved in blood pressure regulation, the renin–angiotensin–aldosterone system (RAAS) contributes to long-term vascular resistance and volume homeostasis through the actions of renin, angiotensin II (Ang II), and aldosterone. RAAS activation is clinically relevant to HTN and renal disease, and therapies targeting this pathway remain central to blood pressure management [3, 4].

**Fig. 1:**
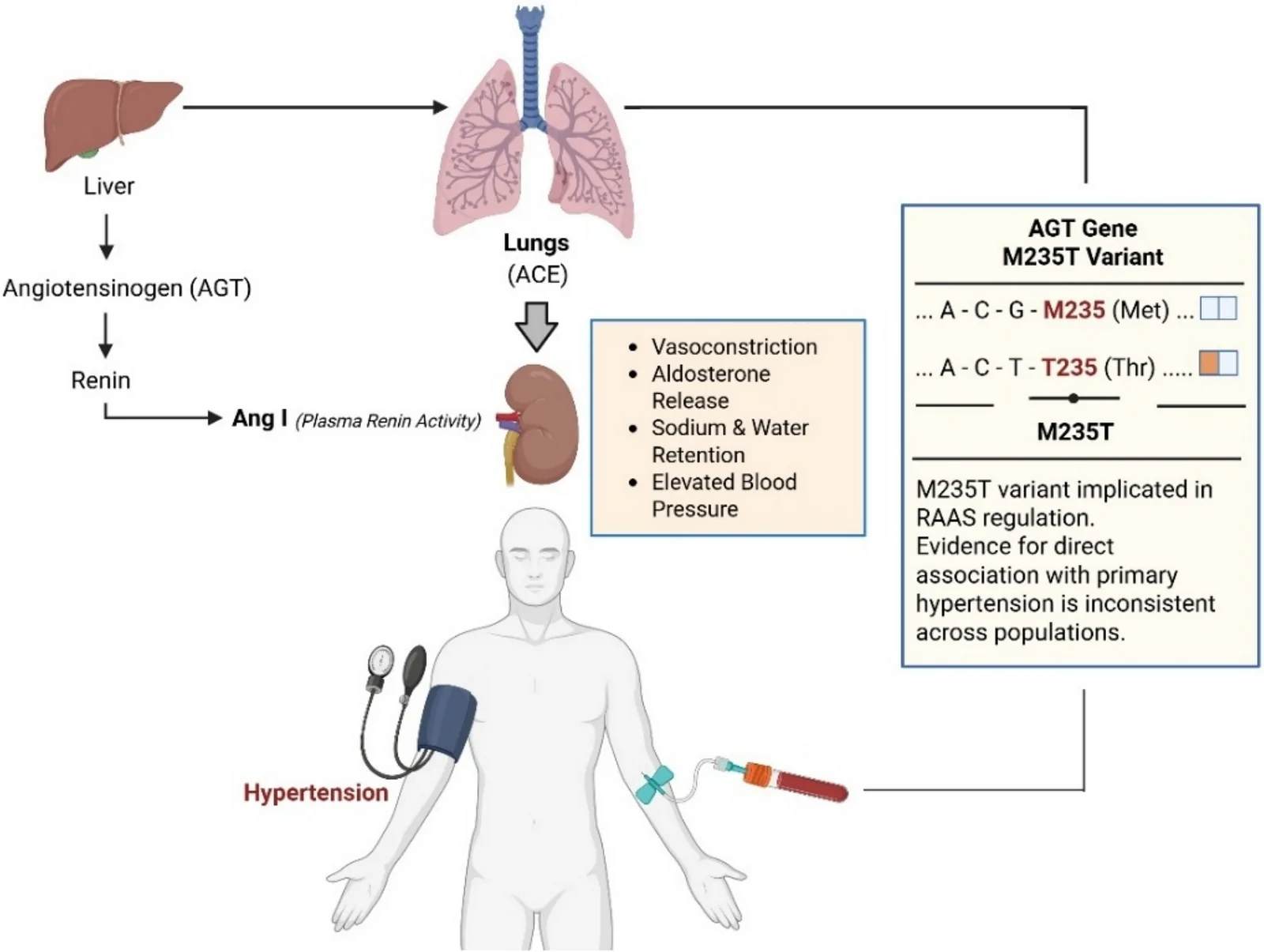
Schematic illustration of the Renin–Angiotensin–Aldosterone System (RAAS) and potential rs699 (M235T) impact (Created in BioRender: https://BioRender.com/qu2tpxm)

The AGT rs699 single nucleotide polymorphism (SNP), commonly referred to as M235T, has been implicated in RAAS-mediated blood pressure regulation [5, 6] (Fig. 1). Reported effects on plasma angiotensinogen concentrations and RAAS activity suggest a potential contribution to elevated blood pressure and vascular remodelling [7]; however, associations with primary HTN remain inconsistent across populations [5, 6, 8, 9]. This inconsistency is particularly relevant in under-studied groups, including older black South Africans, among whom HTN and uncontrolled blood pressure contribute substantially to cardiovascular disease burden [10, 11]. Despite the high burden of HTN in older adults [12, 13], data linking AGT genetic variation to RAAS-related markers such as ACE, Ang I, and Ang II in elderly populations remain limited.

The effect of rs699 may vary with age because age-related RAAS changes, cumulative environmental exposures, and comorbidities can modify genotype–phenotype relationships [9, 14]. Evaluating this variant in older adults may therefore improve cardiovascular risk stratification and inform therapeutic response [15].

Evidence increasingly supports investigation of genetic determinants of blood pressure control and cardiovascular health in older populations. For example, Simino et al. [16] demonstrated age-dependent genetic influences on systolic, diastolic, mean arterial, and pulse pressure through a large-scale consortium-based analysis, while Freire et al. [14] highlighted the relevance of renin–angiotensin–aldosterone system polymorphisms to blood pressure control in hypertensive older adults. Together, these studies strengthen the rationale for examining genetic variation as a contributor to blood pressure regulation and cardiovascular risk in ageing populations [14, 16, 17].

Studies in the same community of the present study have reported elevated cardiovascular risk among older adults [18–21]. Accordingly, this study evaluated the associations of the rs699 SNP with ACE, Ang I, Ang II, BMI, BP and gender in an older black population from Sharpeville, South Africa.

## Methodology and design

### Sample and setting

Ethical approval was granted by the Human Research Ethics Committee (Medical) of the University of the Witwatersrand, Johannesburg (M190669), and all procedures were conducted in accordance with the Declaration of Helsinki and applicable international ethical standards for biomedical research. This exploratory study was embedded within an ongoing longitudinal investigation of an older adult community that has been followed periodically by the principal investigators (Prof. C.J. Grobler and Prof. W.H. Oldewage-Theron) since 2008. Participants were recruited from the only day-care centre in Sharpeville. The inclusion criteria were age ≥60 years, voluntary attendance at the centre, residence in Sharpeville, and provision of informed consent. Individuals who could not complete the informed-consent process, including those with dementia, or who were unable to complete the required study procedures were excluded. Samples were collected during the morning to standardise the collection period. Medication-intake information could not be obtained with sufficient reliability and was therefore not included in the analysis. The final sample consisted of 48 Black South African participants who were either pensioners or unemployed. Sample size was constrained by available funding, the cost of laboratory analyses, and attrition related to advanced age, frailty, illness, or incomplete study procedures.

### Blood collection

A phlebotomist registered with the Health Professions Council of South Africa (HPCSA) collected fasting blood samples from the participants. To ensure confidentiality and anonymity, each participant was assigned a unique study number, which was used for all documentation and blood collection tubes throughout the study. After collection, the vacutainer tubes were gently mixed to achieve proper distribution of the anticoagulants and were immediately centrifuged to separate the plasma, which was then stored at -20°C until further analysis.

### DNA Extraction & SNP Genotyping

Genomic DNA was extracted from peripheral blood samples using the Quick-DNA™ Miniprep DNA purification kit (Zymo Research, Irvine, CA, USA), according to the manufacturer’s protocol. DNA concentration was quantified using the NanoDrop® 2000 spectrophotometer (NanoDrop Technologies, Wilmington, DE, USA), and purity was assessed using the A260/A280 ratio and 1% agarose gel electrophoresis; an A260/A280 ratio of approximately 1.8 was considered indicative of acceptable DNA purity.

Genotyping was performed using the Agena Bioscience MassARRAY system, which applies single-base extension or cleavage chemistry coupled with matrix-assisted laser desorption/ionization-time-of-flight mass spectrometry. The rs699 variant was selected because of its reported association with RAAS activity and HTN [5, 6]. ClinVar entries in the National Center for Biotechnology Information (NCBI) database classify this variant largely as having uncertain significance or conflicting interpretations of pathogenicity, supporting further evaluation in vulnerable elderly populations. Primer/probe synthesis was conducted at Inqaba Biotech (Pretoria, South Africa).

### RAAS parameters—enzyme-linked immunosorbent assays

Standardized enzyme-linked immunosorbent assay (ELISA) kits were used to quantitatively determine the concentrations of Ang I (Immuno-Biological Laboratories Co., Ltd. (IBL) (www.ibl-international.com)), Ang II, and ACE (abcam® (www.abcam.com)). A microplate well coated with an anti-human antibody (Ang I, Ang II, or ACE) was used. Samples or standards were added to the well, where the specific protein (Ang I, Ang II, or ACE) in plasma or serum bound to the anti-human antibody. A biotin-conjugated anti-human antibody (Ang I, Ang II, or ACE) was then added to form a sandwich complex. After incubation, all unbound proteins were washed away, and Streptavidin-Horseradish Peroxidase (HRP) was added, which also bound to the complex. After incubation, another wash removed all unbound Streptavidin-HRP. Tetramethylbenzidine (TMB), a substrate solution reactive with HRP, was used as a chromogen. The color reaction, directly proportional to the quantity of the protein present, developed. The reaction was stopped by the addition of 1M phosphoric acid. Absorbance was measured at 450 nm and compared to standard reference values.

### Anthropometric measurements

Weight was measured in duplicate using two Philips electronic bathroom scales (model HF350), and height was measured with a Scales 2000 portable stadiometer. Measurements were taken on a firm, level surface with participants barefoot and wearing minimal clothing. Height was recorded from the floor to the crown of the head with participants standing upright, heels together, arms at their sides, and the stadiometer headpiece lowered to touch the head. Weight and height were recorded to the nearest 0.1 kg and 0.1 cm, respectively (Table 1, Table 2). BMI was calculated as weight/height2 (kg/m2) [22, 23], and participants were classified according to WHO BMI categories: those with a BMI less than 16.0 were deemed severely underweight, while those with a BMI between 16.0 and 18.4 were classified as underweight. A normal weight was identified in individuals with a BMI ranging from 18.5 to 24.9. Participants falling within the overweight category had a BMI from 25.0 to 29.9. Additionally, those with a BMI between 30.0 and 34.9 were classified as Obese Class I (moderately obese), while those with a BMI from 35.0 to 39.9 were categorized as Obese Class II (severely obese). Lastly, individuals with a BMI of 40.0 or greater were classified as Obese Class III (morbidly obese).

**Table 1:** Descriptive statistics of participants and parameters

Variable	Mean ± SD	Median	Min–Max	Reference Range	Source
ACE (ng/mL)	186.0 ± 306.4	128.3	77.9–2230.2	130–450 ng/mL	[28, 29]
Ang I (ng/mL)	40.37 ± 13.76	47.16	1.23–49.72	0.2–60 ng/mL	[30]
Ang II (pg/mL)	76.80 ± 98.34	48.11	-21.38–450.61	10–60 pg/mL	[31]
SBP (mmHg)	157.83 ± 20.66	157.0	123.5–217.0	SAHS 2019 guidelines	
DBP (mmHg)	97.13 ± 13.12	98.5	69.0–131.5		
BMI (kg/m^2^)	29.91 ± 5.23	29.62	16.16–40.35	WHO Adult BMI Categories

**Table 2:** Associations between the rs699 SNP and selected RAAS parameters using Firth’s penalized logistic regression

Parameter	Firth OR	95% Confidence Interval	*p*-value
ACE	0.9879	0.9560 – 1.0208	0.4663
Ang I	0.9798	0.9098 – 1.0553	0.5904
Ang II	1.0013	0.9902 – 1.0125	0.8198
BP	0.9895	0.9299 – 1.0529	0.7384
BMI	1.0830	0.8513 – 1.3777	0.5164
Gender	0.8824	0.0328 – 23.7175	0.9406

### Blood pressure (BP)

The subjects were requested to sit quietly for at least 5 minutes in a chair with a back support, feet on the floor and arm supported at feet level before the measurements. Blood pressure was measured using the Tensoval® duo control automated upper-arm monitor, with an appropriately sized cuff applied securely in accordance with the manufacturer’s instructions. Two measurements were obtained on each of two separate days, and the mean systolic and diastolic blood pressure values were calculated for analysis. This device was selected because it combines oscillometric measurement with auscultatory detection of Korotkoff sounds, providing an automated approach suitable for standardized data collection while retaining a measurement principle used in routine clinical practice. Its accuracy in adults was evaluated against sequential same-arm measurements obtained with a mercury sphygmomanometer; the device achieved an A/A grade under the British Hypertension Society protocol and met the European Society of Hypertension International Protocol criteria, supporting its use for clinical, home and field-setting blood pressure measurement [24].

HTN was defined according to the SAHS guidelines at 140/90 mmHg. These guidelines were used to further divide the elderly population into the categories; normal and HT grade (1 – 3) (Table 3).

**Table 3:** Classification of patient systolic blood pressure readings according to the SAHS categories

Category	SBP Criterion (mmHg)	Number of readings	Percentage of readings
Normotension	<140	12	24.5%
Grade 1 hypertension	140–159	15	30.6%
Grade 2 hypertension	160–179	17	34.7%
Grade 3 (Severe) hypertension	≥180	5	10.2%

### Data analysis and modelling

The rs699 genotypes, along with data on age, ACE, Ang I, Ang II, BMI, gender and BP readings, were captured in a Microsoft Excel spreadsheet. The raw data were thoroughly cross-checked against the captured data to ensure accuracy. The data analyses and visualisations were conducted using IBM SPSS Statistics® version 27 and Python (Version 3.14) (Python Software Foundation, 2026).

For the rs699 analysis, raw genotype calls were standardised as diploid categories: G calls were coded as GG, AG calls were retained as heterozygous genotypes, AA was retained as the alternative homozygous category despite the absence of observed carriers, and null calls were designated as missing and excluded from allele-frequency and Hardy–Weinberg equilibrium (HWE) calculations. Frequencies were represented by p and q, where p + q = 1, and the corresponding expected genotype frequencies under HWE were estimated as p2, 2pq, and q2. Genotype-level quality control included the exclusion of samples with call rates below 95% to minimise potential bias attributable to excessive missingness. Variants with a minor allele frequency below 0.05 were excluded because low-frequency alleles may yield unstable estimates and reduced statistical power [25]. Concordance between observed and HWE-expected genotype distributions was evaluated using an exact test.

Associations between the rs699 SNP and RAAS-related markers were evaluated using Firth’s penalized logistic regression to reduce small-sample bias arising from the low prevalence of the rs699 AG genotype and the limited sample size [26, 27]. Genotype was modelled as a three-level categorical variable. To assess the robustness of the findings under sparse genotype distributions, sensitivity analyses were conducted using four model specifications: standard maximum-likelihood logistic regression, a baseline Firth model, a Firth model after outlier exclusion, and a Firth model fitted to the imputed dataset. Associations involving dichotomized outcome variables were summarized as odds ratios.

## Results

The study population had a mean age of 73 years and was predominantly female (81.2%), with only a few males (16.7%). The average concentration of the DNA yield was 32.81ng/µL. The rs699 SNP was detected in our sample with a skewed distribution. Most of the participants (93.8%) were carriers of the GG genotype, 4.2% of the AG genotype and the SNP was not detected in one participant. Genotyping was successful in 97.92% of samples, indicating a low proportion of missing genotype data. The observed genotype distribution conformed to Hardy–Weinberg equilibrium, with no significant deviation detected by either the chi-square goodness-of-fit test (χ2 = 0.0222, df = 1, p = 0.8815) or Haldane’s exact test (p = 1.0000). Collectively, these findings indicate robust genotyping performance and support the inclusion of rs699 in subsequent analyses. The Hardy–Weinberg equilibrium testing and genotyping quality metrics are shown in Supplementary Tables 1 and 2.

Across the ACE, Ang I, and Ang II assays, intra-assay coefficients of variation (CVs) were <8%, with all values remaining below 10%, whereas inter-assay CVs were <10%, with all values remaining below 12%. These findings demonstrate consistently high within-run and between-run precision across all three assays. The ACE concentrations were below the reference range in most participants (64.6%), although one markedly elevated value influenced the overall distribution, while Ang I concentrations remained within the stated reference range among those measured. Ang II was elevated in 64.6% of participants. The cohort showed a high blood pressure burden, with a mean systolic BP of approximately 158 mmHg and most participants classified as hypertensive (Table 3). Adiposity was also substantial, with 17 participants (35.4%) classified as overweight, 13 (27.1%) as obesity class I, 8 (16.7%) as obesity class II, and 1 (2.1%) as obesity class III; the mean BMI was approximately 29.9 kg/m^2^.

### Logistic regression model

None of the evaluated parameters (ACE, Ang I, Ang II, BP, BMI, or gender) was significantly associated with the rs699 SNP in the univariate, multivariable, or Firth-penalized regression models. The predominance of the GG genotype in the study population may have reduced the statistical power to detect modest effect sizes.

### Sensitivity analysis

Sensitivity analyses yielded consistent findings across all four model specifications, with odds-ratio point estimates remaining close to the null value of 1.0 (range: 0.9691–1.0957). All *p*-values exceeded 0.05, indicating no statistically significant association between the evaluated clinical parameters and the rs699 SNP in this cohort.

## Discussion

This exploratory study examined associations between the AGT rs699 SNP, selected RAAS parameters (ACE, Ang I, and Ang II), BMI, BP and gender in an elderly Black population from Sharpeville, South Africa. Approximately 75% of participants had Grade 1–3 HTN, including nearly 44% with Grade 2–3 HTN, consistent with the substantial cardiovascular risk burden previously reported in this community [18–21]. This high prevalence may reflect the combined effects of ageing, excess adiposity, and altered cardiovascular regulation [32, 33]. Age-related chronic low-grade inflammation (“inflammaging”) may further promote endothelial dysfunction, vascular stiffening, and elevated BP [34]. Together with previously documented nutritional and lifestyle challenges, these factors may contribute to the cohort’s cardiovascular risk profile.

ACE concentrations were generally below reference ranges, consistent with previous findings [35]. Given the central role of ACE in BP regulation and fluid balance, these concentrations may reflect age-related changes in RAAS activity, genetic variation, or antihypertensive treatment, particularly ACE inhibitor use [36, 37]. Because medication use could not be assessed, the low ACE concentrations should be interpreted cautiously and not attributed solely to pathological dysfunction.

Angiotensin II concentrations varied considerably among participants, consistent with previous studies [38–41]. This variability may reflect complex RAAS regulation in older adults: ageing is often associated with reduced plasma renin activity and low-renin HTN, whereas tissue RAAS activity may remain elevated and promote vascular remodelling despite lower circulating Ang II. Conversely, systemic RAAS activation may increase circulating Ang II in some individuals [38–41]. Some female participants had higher mean Ang II concentrations than males; however, substantial variability and the small male sample preclude firm interpretation. Furthermore, circulating ACE, Ang I and Ang II may not reliably represent RAAS activity because systemic concentrations do not necessarily reflect tissue-level signalling. This distinction is relevant to HTN and ageing, in which local autocrine and paracrine RAAS pathways may operate independently of the endocrine system and contribute to organ remodelling, inflammation, and oxidative stress. Assessment of both circulating and tissue RAAS activity may therefore provide a more robust mechanistic interpretation of disease-related changes [37].

Overweight or obesity affected approximately 81% of participants, with nearly 46% classified as obese. Female participants had a higher mean BMI, whereas males had a higher mean systolic BP. In older adults, obesity-related HTN is linked to age-associated visceral fat accumulation, which promotes chronic inflammation, arterial stiffness, insulin resistance, renal compression, impaired sodium excretion and neurohumoral activation [42, 43]. Thus, the high prevalence of excess adiposity may have contributed substantially to the cohort’s HTN burden.

The rs699 SNP is a missense variant in the AGT gene on chromosome 1q42–q43 that causes a methionine-to-threonine substitution and has been widely investigated in RAAS-mediated BP regulation [44, 45]. It may influence plasma angiotensinogen concentrations and HTN risk, although associations vary across populations and study designs [7, 9]. This heterogeneity may reflect population-specific genetic backgrounds, environmental exposures, and interactions with other variants [6, 46]. In the present study, marked genotype imbalance limited statistical power, and the rs699 SNP was not significantly associated with ACE, Ang I, Ang II, BP, BMI or gender. This finding is consistent with evidence that the rs699 (M235T) alone is unlikely to be a universal predictor of HTN or RAAS activity and that its effects depend on broader genetic and environmental contexts [47, 48]. Nevertheless, the result should be interpreted cautiously.

## Limitations

Interpretation is limited by the small sample and statistical power, gender and genotype imbalances, analysis of a single SNP, absence of a control group, and unavailable medication data. In addition, the ELISA kits provided analytical detection ranges rather than universal clinical reference intervals, so the ranges reported in Table 1 were used. Accordingly, the results are population-specific and exploratory: no statistically significant associations were detected, but potentially relevant effects cannot be excluded.

## Conclusion

In this small exploratory cohort of older adults, no statistically significant associations were detected between rs699 and ACE, Ang I, Ang II, BP, BMI or gender; BMI showed a borderline sex-related difference, with higher values in females. These findings are hypothesis-generating and do not exclude potentially meaningful effects, warranting adequately powered, genotype-balanced studies in diverse African populations.

## Supplementary Information

Below is the link to the electronic supplementary material.

Supplementary file1 (DOCX 20 KB)

## Data Availability

De-identified data or restricted-access raw files are available to qualified researchers upon approval from the Human Research Ethics Committee (Medical) of the University of the Witwatersrand.
