# Supplementary material for: The AGT M235T variant and renin–angiotensin–aldosterone system parameters in a predominantly hypertensive elderly black South African cohort: a hypothesis-generating descriptive case series
Source: Mol Biol Rep. 2026 Oct 23;53(1):1790. doi: 10.1007/s11033-026-12994-2 (PMC13694406; doi:10.1007/s11033-026-12994-2)
Supplement: Supplementary file 1 [file 11033_2026_12994_MOESM1_ESM.docx]

**Supplementary Table 1: Genotyping Quality Metrics**

| **Metric** | **Value** |
| --- | --- |
| Total Samples | 48 |
| Successfully Genotyped Samples | 47 |
| Missing / Uncalled Samples (Null) | 1 |
| Sample Call Rate | 97.92% |
| Missingness Rate | 2.08% |
| Total Alleles Analyzed (*2N*) | 94 |

**Supplementary Table 2: Hardy–Weinberg Equilibrium Test**

| **Genotype Category** | **Observed Count (*O*)** | **Expected Count (*E*)** | **Deviation (*O-E*)** |
| --- | --- | --- | --- |
| GG (Homozygous Major) | 45 | 45.0213 | -0.0213 |
| AG (Heterozygous) | 2 | 1.9574 | +0.0426 |
| AA (Homozygous Minor) | 0 | 0.0213 | -0.0213 |

**Supplementary Table 3: Sensitivity analysis**

| **Parameter** | **Standard MLE Logistic Model** | **Firth Baseline Model** | **Firth Outlier-Filtered Model** | **Firth Imputed Data Model** |
| --- | --- | --- | --- | --- |
| ACE | 0.9671 (0.9070 – 1.0311)  *p* = 0.307 | 0.9879 (0.9560 – 1.0208)  *p* = 0.466 | 0.9691 (0.9161 – 1.0252)  *p* = 0.276 | 0.9879 (0.9560 – 1.0208)  *p* = 0.466 |
| AngI | 0.9938 (0.9010 – 1.0962)  *p* = 0.901 | 0.9798 (0.9098 – 1.0553)  *p* = 0.590 | 0.9856 (0.9125 – 1.0645)  *p* = 0.704 | 0.9863 (0.9221 – 1.0551)  *p* = 0.686 |
| AngII | 0.9970 (0.9789 – 1.0155)  *p* = 0.752 | 1.0013 (0.9902 – 1.0125)  *p* = 0.820 | 1.0028 (0.9892 – 1.0167)  *p* = 0.685 | 1.0013 (0.9902 – 1.0125)  *p* = 0.820 |
| BP | 0.9789 (0.9040 – 1.0601)  *p* = 0.600 | 0.9895 (0.9299 – 1.0529)  *p* = 0.738 | 0.9911 (0.9288 – 1.0577)  *p* = 0.781 | 0.9898 (0.9304 – 1.0530)  *p* = 0.738 |
| BMI | 1.0957 (0.8239 – 1.4571)  *p* = 0.530 | 1.0830 (0.8513 – 1.3777)  *p* = 0.516 | 1.0945 (0.8507 – 1.4082)  *p* = 0.485 | 1.0805 (0.805 – 1.3721)  *p* = 0.523 |

*Values represent Odds Ratio (95% Confidence Interval) and p-value*
